# Habitat and Seasonality Affect Mosquito Community Composition in the West Region of Cameroon

**DOI:** 10.3390/insects11050312

**Published:** 2020-05-15

**Authors:** Marie Paul Audrey Mayi, Roland Bamou, Borel Djiappi-Tchamen, Albin Fontaine, Claire L. Jeffries, Thomas Walker, Christophe Antonio-Nkondjio, Anthony John Cornel, Timoléon Tchuinkam

**Affiliations:** 1 Vector Borne Diseases Laboratory of the Research Unit of Biology and Applied Ecology (VBID-RUBAE), Department of Animal Biology, Faculty of Science of the University of Dschang, P.O. Box 067 Dschang, Cameroon; bamou2011@gmail.com (R.B.); borel_tchamen@yahoo.com (B.D.-T.); timotchuinkam@yahoo.fr (T.T.); 2 Laboratoire de Recherche sur le Paludisme, Organisation de Coordination pour la lutte Contre les Endémies en Afrique Centrale (OCEAC), P.O. Box 288 Yaoundé, Cameroon; antonio_nk@yahoo.fr; 3 Unité Parasitologie et Entomologie, Département Microbiologie et maladies infectieuses, Institut de Recherche Biomédicale des Armées (IRBA), 19-21 Boulevard Jean Moulin, 13005 Marseille, France; albinfont@gmail.com; 4 Aix Marseille Univ, IRD, SSA, AP-HM, UMR Vecteurs—Infections Tropicales et Méditerranéennes (VITROME), 13005 Marseille, France; 5 IHU Méditerranée Infection, 13005 Marseille, France; 6 Department of Disease Control, Faculty of Infectious and Tropical Diseases, London School of Hygiene and Tropical Medicine, London WC1E 7HT, UK; Claire.jeffries@lshtm.ac.uk (C.L.J.); thomas.walker@lshtm.ac.uk (T.W.); 7 Department of Entomology and Nematology, Mosquito Control Research Laboratory, University of California at Davis, Parlier, CA 93648, USA; ajcornel@ucanr.edu

**Keywords:** emerging vector-borne diseases, arboviruses, mosquito-vectors, urbanisation, Dschang, Cameroon

## Abstract

To identify potential sylvatic, urban and bridge-vectors that can be involved in current or future virus spillover from wild to more urbanised areas, entomological field surveys were conducted in rural, peri-urban and urban areas spanning the rainy and dry seasons in western Cameroon. A total of 2650 mosquitoes belonging to 37 species and eight genera were collected. Mosquito species richness was significantly influenced by the specific combination of the habitat type and the season. The highest species richness was found in the peri-urban area (S = 30, Chao1 = 121 ± 50.63, ACE = 51.97 ± 3.88) during the dry season (S = 28, Chao1 = 64 ± 25.7, ACE = 38.33 ± 3.1). *Aedes (Ae.) africanus* and *Culex* (*Cx.) moucheti* were only found in the rural and peri-urban areas, while *Cx. pipiens* s.l. and *Ae. aegypti* were only found in the urban area. *Cx. (Culiciomyia)* spp., *Cx. duttoni* and *Ae. albopictus* were caught in the three habitat types. Importantly, approximately 52% of the mosquito species collected in this study have been implicated in the transmission of diverse arboviruses. This entomological survey provides a catalogue of the different mosquito species that may be involved in the transmission of arboviruses. Further investigations are needed to study the vectorial capacity of each mosquito species in arbovirus transmission.

## 1. Introduction

Mosquitoes are vectors of many pathogens that threaten the health of humans and animals worldwide [1,2,3,4,5,6]. Patterns of mosquito species composition and abundance are influenced by environmental factors, such as temperature, humidity and the availability of suitable breeding sites [7], which usually vary across seasons. Seasonality also directly affects larval development and mosquito adult abundance and, therefore, indirectly affects disease transmission [8].

Global climate change, increased international travel, deforestation, urbanisation and the large-scale use of pesticides (mostly organochlorines, such as DDT (Dichlorodiphenyltrichloroethane)) are the main drivers of vector-borne diseases outbreaks, emergence and re-emergence [3,9,10,11,12,13]. In Malaysia, the emergence of the Nipah virus has been linked to the intensification of agricultural activities [14]. In the eastern United States, forest fragmentation and urbanisation led to reduced host diversity, allowing disease-competent rodent hosts to dominate the community, contributing to the emergence of Lyme disease [15]. In the southeastern part of Cote d’Ivoire, where large parts of the rainforest have been converted into oil palm plantations, several outbreaks of yellow fever and dengue have been documented [16]. Around 60 examples of linkage between deforestation and land-use changes and increases in mosquito populations and malaria risk were noted by Yasuoka and Levins [17]. In Cameroon, an increase in the prevalence of avian haemosporidian parasites in some bird communities [18], and the abundance of female mosquitoes, following habitat fragmentation has been reported [18,19].

Urbanisation is one of the main causes of deforestation, resulting in the alteration of the natural environment to make it more suitable for human populations and to accommodate both the growth of the local population and people moving from rural areas to cities [20]. However, urbanisation through the expansion of roads and infrastructure creates suitable habitats for the proliferation of anthropophilic mosquito vectors of diseases of public health importance [21,22]. Additionally, urbanisation reduces the biodiversity and richness of sylvatic/forest mosquito species, subsequently increasing the abundance of species that can adapt to urban ecological niches, such as *Ae. aegypti* and *Cx. pipiens* s.l., and increasing the risk of human disease transmission [23,24]. The introduction of species into new habitats provides opportunities for novel pathogens to infect human populations, which could lead to the emergence and spread of new diseases [25,26]. For example, the transmission of yellow fever virus (YFV) to human populations from sylvatic cycles was seen in South America [27]. Within the jungle (sylvatic) cycle, YFV is transmitted by *Haemagogus, Sabethes* and *Aedes* mosquitoes to monkeys in the rainforest canopy. After logging and land clearing, mosquitoes followed the canopy edge to the ground where they fed on, and infected, humans [27,28]. To minimise global disease emergence, it is crucial to understand the factors that influence disease emergence and how to control these factors. However, the few studies that have assessed the impact of deforestation and urbanisation on mosquito vector communities in Cameroon mostly focused on malaria vector populations [29,30] whilst abundance trends and diversity patterns for all other mosquito groups have been largely neglected or undocumented [18,31].

In tropical forest regions of Africa, there are numerous mosquito-borne zoonotic viruses, such as Semliki Forest, Sindbis, Spondweni, Uganda S, o’nyong-nyong, Bwamba, Bunyamwera and Shuni viruses, which currently have not been shown to result in major disease symptoms in humans and animals [32]. The impact of deforestation on local mosquito species abundance and diversity could lead to more severe pathogenic symptoms in humans for these circulating viruses [32]. Examples of previously benign viruses circulating for millennia in African forests that have, in recent times, caused global human disease epidemics because of host and vector switching due to minor viral genome mutations, including Zika (ZIKV) and chikungunya (CHIKV) viruses [33,34]. In Cameroon, Braack et al. [35] reviewed many arboviruses, which include dengue virus (DENV), Ntaya virus (NTAV), Spondweni virus (SPOV), Yaounde virus (YAOV), YFV, CHIKV, Semliki Forest virus (SFV), Sindbis virus (SINV), Rift Valley fever virus (RVFV), Bunyamwera virus (BUNV), Bwamba virus (BWAV) and Ilesha virus (ILEV).

In addition to the many human cases of arboviruses reported in the different regions of Cameroon; South [36,37], Littoral [38], Centre [39], Southwest [40], Northwest [41], West, Far North and Adamaoua [42], the country is undergoing a rapid increase in urbanisation that is impacting the population dynamics of mosquito species and, subsequently, the risk of arbovirus transmission to humans. Dschang is one such city in Cameroon which has experienced over the past years, a modification of its natural environment. The rapid and spontaneous urbanisation in and around the city, through the construction of roads and buildings (while there is a lack of infrastructure for sanitation and drainage), as well as the colonisation of lowland areas for agricultural activities, have favoured the development and installation of mosquitoes in the city. These anthropogenic changes are considered to have major influence on the epidemiology of vector borne diseases [43,44,45].

In the face of these threats, an assessment of mosquito species’ composition, including mosquito abundance and species diversity, are required to develop projections and models to predict likely areas in which arbovirus outbreaks could occur [32]. Entomological surveys can then provide important insights into the influence of urban planning and prevention on arboviral epidemics. In the western part of Cameroon, very few studies have examined the circulation of arboviruses in human populations and the presence of mosquito vectors [42,46,47]. Given the lack of studies looking at the mosquito composition after urbanisation in western Cameroon, we undertook an entomological survey to identify potential sylvatic, urban and bridge-vector species that could potentially play a role in current or future virus spillover from wild to more urbanised areas. Furthermore, we assessed the effect of landscape anthropisation following a transect of urbanisation (from rural to urban settings) on mosquito species abundance, composition and distribution across seasons and how this may influence the potential risk of arboviral infections in the area.

## 2. Materials and Methods

### 2.1. Description of the Study Sites

**Figure 1:**
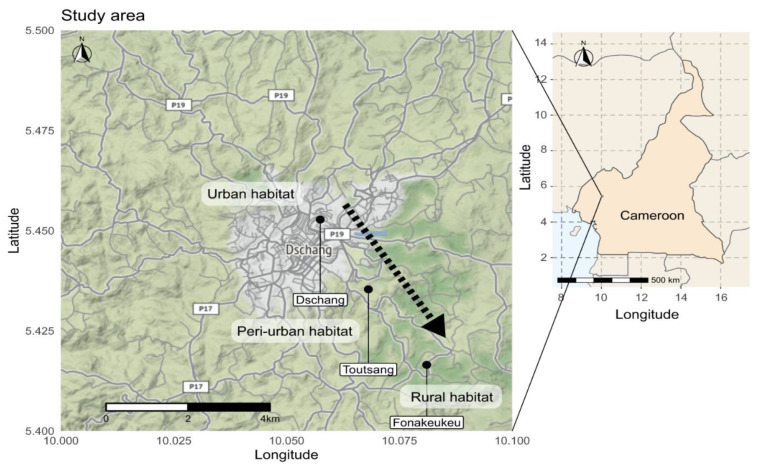
Map of collection sites in Dschang, West-Cameroon. Mosquito sampling was done in three habitats along a transect of urbanisation.

This study was carried out in the Dschang sub-division within the Menoua Division in the West region of Cameroon. Dschang is situated at 1500 m above sea level and is surrounded by many villages. Three types of habitats were selected based on the degree of urbanisation and the following characteristics: number of persons per km^2^ (human population size), presence/absence of infrastructure and the type of vegetation. The rural and peri-urban habitats were located in Fonakeukeu (05°24′73″ N, 010°04′79″ E), and Toutsang (05°25′65″ N, 010°04′05″ E) villages, respectively, while the urban habitat was located in the town of Dschang (05°16′87″ N, 009°58′42″ E) (Figure 1).

**Figure 2:**
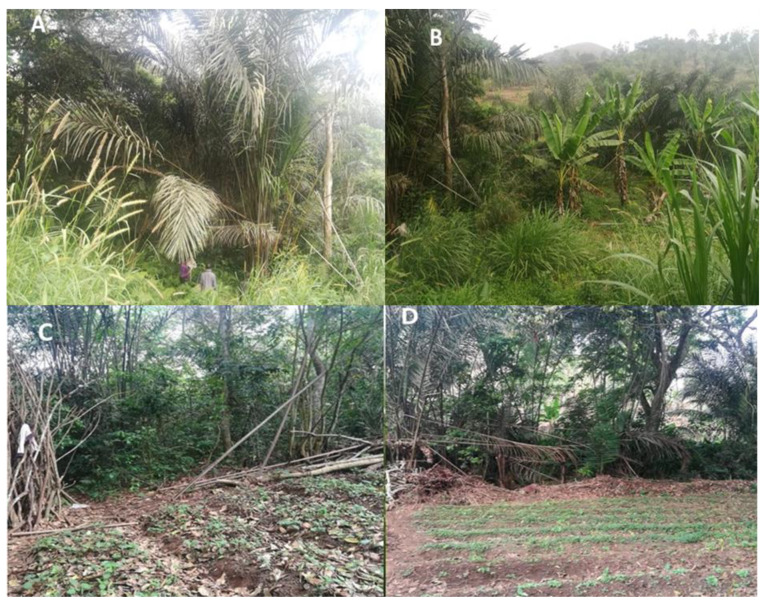
Rural (Fonakeukeu) and peri-urban (Toutsang) sites with raffia palm bushes (**A**), ‘Toutsang’ surrounded by a banana farm (**B**) and other cultivated areas in ‘Fonakeukeu’ (**C**) and ‘Toutsang’ (**D**).

All study sites are located in the highland area of western Cameroon that exhibits a sub-tropical climate characterised by two seasons; a dry season of four months (from mid-November to mid-March) and a rainy season of eight months (mid-March to mid-November). The annual average rainfall and temperature of this region are 321 mm and 21.6 °C, respectively [42]. An urban area (Dschang) was defined as a location with more than 1000 persons per km^2^ [44]. Dschang contains shrub-like vegetation, an important hydrographic network and many artificial breeding sites that might provide ideal environmental conditions for urban mosquito species. A peri-urban area was defined as a location with 250–1000 persons per km^2^, while a rural area was defined as a location with fewer than 250 persons per km^2^ [44]. The peri-urban (Toutsang) and rural (Fonakeukeu) sites in our study are located approximately 2 km and 5 km, respectively, from the urban habitat (Dschang). Raffia palm bushes are common in Toutsang and Fonakeukeu and are found along the valleys and streams, which might offer natural year-round breeding sites for sylvatic mosquito species (Figure 2).

### 2.2. Mosquito Sampling, Identification and Preservation

Entomological field surveys were conducted at each site (rural, peri-urban, urban) during the rainy season (March–September, 2019) and the dry season (November, 2019–February, 2020). A four-day mosquito collection survey was undertaken at each site during each season by collecting immature stages in available breeding sites (abandoned tyres, metallic and plastic containers, gutters, stagnant water pools, riverbeds or floor pools) and using sweep nets to catch mosquito adults resting on vegetation. Immature collections and sweep netting were done during a four-hour period each day by four people at each site. In all sites, the sampling surface covered relied on the presence of breeding and resting sites of mosquitoes; in rural and peri-urban sites, collections were focused on the raffia palm bushes, while in the urban area, mosquito collections were mainly done in the town centre or in quarters. The types of breeding sites and their characteristics were recorded (when possible) and are being considered in another paper. Immature stages were reared to adults in the Vector Borne Diseases Laboratory of the Research Unit of Biology and Applied Ecology (VBID-RUBAE) at the University of Dschang before identification. Morphological identification of mosquito adults was done using stereomicroscopes and morphological identification keys [48,49] named according to the stable classification of *Aedini* [50]. Identified mosquitoes were preserved either in silica gel, in 70% ethanol or in RNA later for further molecular analysis for pathogen detection.

### 2.3. Data Analysis

Statistical analysis was performed using the environment for statistical computing and graphics R [51]. Mosquito abundance was defined as the total number of mosquitoes captured, independently of the species. Species richness (S) or number of species or taxa in the community was determined per habitat type and per season. Two estimators of the ‘true’ number of species in each site, Chao1 and ACE (Abundance-base Coverage Estimator), were calculated using the command estimate R in the ‘vegan’ package [52]. Individual-based rarefaction curves for all sites across seasons were constructed from EstimateS software. PCA (Principal Component Analysis) was performed with the FactoMine R package [53] to display the association between mosquito species and habitat types, and mosquito species and seasons. Mosquito abundance across habitats and seasons was treated as count data and analysed using negative binomial regression. A full-factorial negative binomial generalised linear model that included the mosquito species, the habitat and season was fitted to the data. Mosquito species that were represented with fewer than two counts across habitats and season were discarded for analysis. Statistical significance of the predictors’ effects was assessed by comparing nested models for changes in deviances (goodness of fit measure for a model) based on a chi-squared distribution with the R package car [54] using the type III sums of squares method in the presence of significant interaction between predictors. The same procedure was applied per mosquito species. Significance was determined at the threshold of 1.35 × 10^-3^ by applying Bonferroni correction for multiple testing. The R package ggmap [55] was used to create the map of the study area.

## 3. Results

### 3.1. Mosquito Abundance across Habitats and Seasons

**Figure 3:**
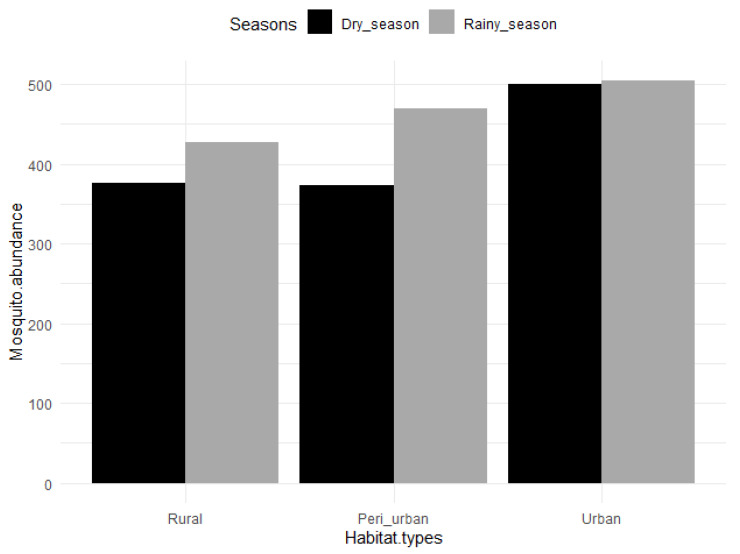
Mosquito abundance across habitats and seasons in Dschang, West-Cameroon.

A total of 2650 mosquitoes (1800 females and 850 males) were collected in the entomological survey (Table 1) comprising 1642 mosquitoes collected as adults, and 1008 mosquitoes as larvae and pupae (immature stage). The immatures were mainly collected in the urban area (*n* = 998) in abandoned tyres, containers (metallic and plastic), gutters and stagnant water pools (Unpublished data). Difficulty in finding breeding sites in peri-urban and rural areas resulted in the low number of immatures collected in riverbeds (*n* = 10). On the other hand, sweep nets were not effective in urban areas with only 6 adult mosquitoes captured compared to the high numbers captured using this method in peri-urban (*n* = 841) and rural (*n* = 795) areas. Of the 2650 mosquitoes, 2389 were morphologically identified to species. The remaining 281 mosquitoes were only identified to genera and subgenera because the specimens belonged to cryptic species complexes or were damaged (i.e., most of their scales were completely rubbed off during the process of collection in the traps). Out of the 2650 mosquitoes, 803 (30.30%) were collected in the rural area, 843 (31.81%) in the peri-urban area, and 1004 (37.89%) in the urban area. When comparing seasons, 1401 (52.87%) mosquitoes were recorded during the rainy season, while 1249 (47.13%) were collected during the dry season (Table 1). Mosquitoes were evenly distributed across seasons in the urban area (*n* = 500, *n* = 504 for dry and rainy seasons, respectively), while more mosquitoes were captured during the rainy season in the rural area (*n*= 376, *n* = 427 for dry and rainy seasons, respectively) and peri-urban area (*n* = 373, *n* = 470 for dry and rainy seasons, respectively) (Figure 3). However, neither habitat type nor seasonality significantly influenced mosquito abundance independently of the mosquito species (*p* > 0.05 according to the negative binomial regression).

### 3.2. Mosquito Species Richness across Habitats and Seasons

At least 37 mosquito species belonging to eight genera were collected. The genus *Aedes* recorded the highest number of species (14 species) closely followed by *Culex* (12 species). The other genera (*Eretmapodites*, *Coquillettidia*, *Uranotaenia, Lutzia* and *Anopheles)* recorded less than four species each (Table 1). Out of the 37 species, *Ae. africanus* were the most abundant (*n* = 1023, 36.6%), followed by *Cx. duttoni* (*n* = 510, 19.25%), *Cx. moucheti* (*n* = 242, 9.13%), *Cx. (Culiciomyia)* spp. (*n* = 218, 8.23%), *Ae. albopictus* (*n* = 192, 7.25%) and individuals of the *Cx. pipiens* complex (*n* = 174, 6.57%) (Table 1).

#### 3.2.1. Habitat Type Effect

There was a significant difference (*p* < 0.05) for mosquito species richness across habitat types. The highest richness was found in the peri-urban area (S = 30, Chao1 = 121 ± 50.63, ACE = 51.97 ± 3.88), while the lowest richness was found in the urban area (S = 11, Chao1 = 11 ± 0.48, ACE = 11.33 ± 1.57) (Table 2).

**Figure 4:**
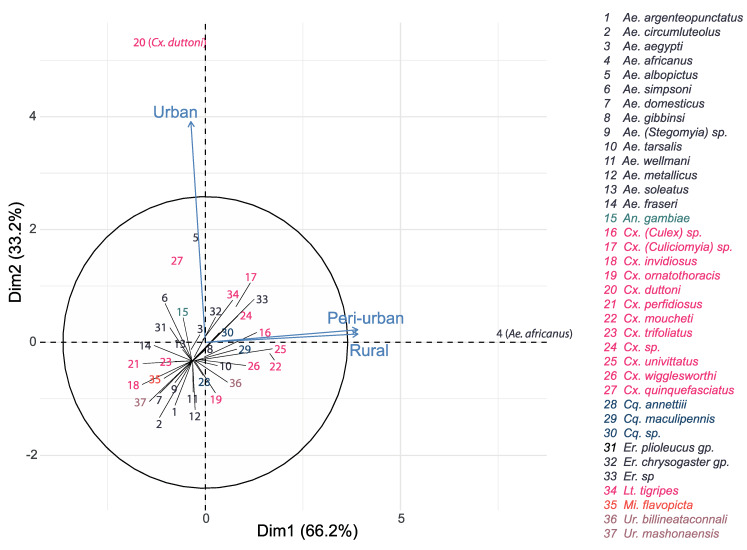
Principal component analysis representing the relationship between mosquito species abundance and habitat types.

The most dominant species in the rural area and peri-urban area were *Ae. africanus* (*n* = 533, *n* = 490, respectively) and *Cx. moucheti* (*n* = 142, *n* = 100, respectively), while *Cx. duttoni* (*n* = 501) dominated the urban area, followed by *Ae. albopictus* (*n* = 184) and *Cx. pipiens* s.l. (*n* = 174) (Table 1 and Figure 4). More importantly, *Ae. africanus* and *Cx. moucheti* were only found in the rural and peri-urban areas, while *Cx. pipiens* s.l. and *Ae. aegypti* were only found in the urban area. (Table 1).

#### 3.2.2. Seasonality Effect

Higher species richness was recorded in the dry season (S = 28, Chao1 = 64 ± 25.7, ACE = 38.33 ± 3.1) rather than in the rainy season (S = 24, Chao1 = 24.6 ± 1.78, ACE = 26.04 ± 2.49) (Table 3). A significant interaction between mosquito species richness and seasons was found (*p* < 0.05).

**Figure 5:**
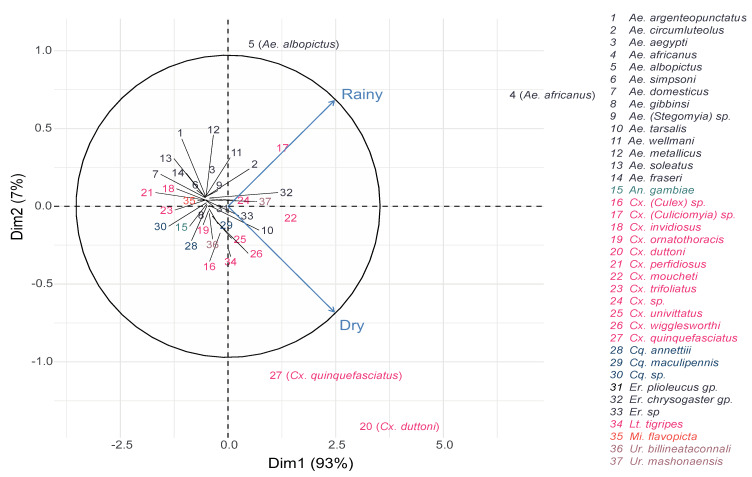
Principal component analysis representing the relationship between mosquito species abundance and seasons.

Overall, *Culex* spp. were more abundant during the dry season, while *Aedes* spp. were most abundant during the rainy season (Figure 5). The most dominant species in both rainy and dry seasons were *Ae. africanus* (*n* = 637 and *n* = 386, respectively) and *Cx. duttoni* (*n* = 199 and *n* = 311, respectively). They were followed by *Ae. albopictus* (*n* = 180) and *Cx*. (*Culiciomyia)* spp. (*n* = 144) in the rainy season, then *Cx. pipiens* s.l. (*n* = 153) and *Cx. moucheti* (*n* = 114) in the dry season (Table 1 and Figure 5). Interestingly, some species, such as *Ae. aegypti*, *Ae. metallicus, Ae. wellmani* and *Ae. Soleatus,* were only captured during the rainy season, while other species, such as *Ae. gibbinsi*, *Cx. ornatothoracis*, *Uranotaenia* spp. and *Mimmomyia* spp. were solely trapped during the dry season (Table 1).

#### 3.2.3. Habitat: Seasonality Effect

**Figure 6:**
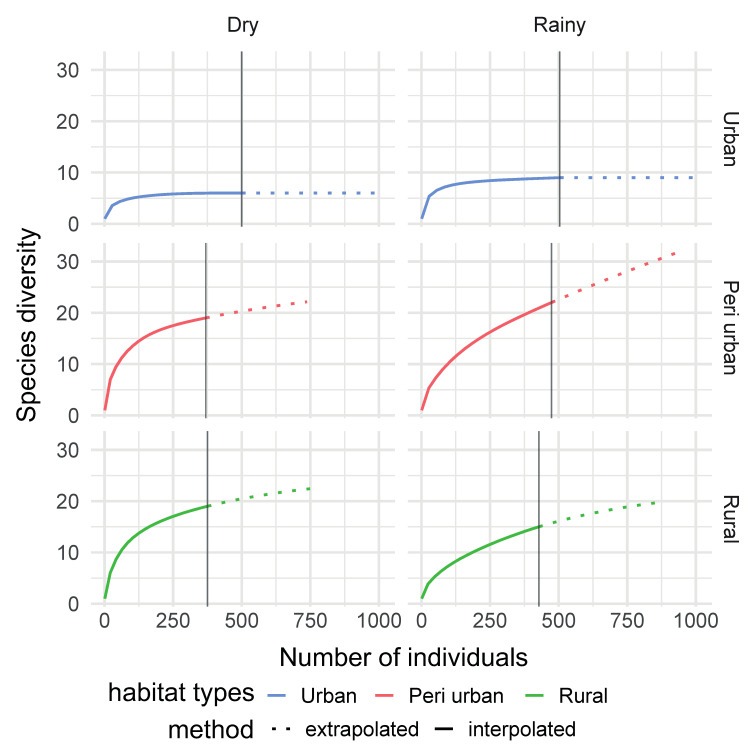
Individual-based rarefaction curves comparing species richness across habitats and seasons in Dschang, West-Cameroon.

A higher number of mosquito species were found during the rainy season in the urban area (S = 9, 6 for rainy and dry seasons, respectively), and peri-urban area (S = 22, 19 for rainy and dry seasons, respectively), while more mosquito species were captured during the dry season in the rural area (S = 15, 19 for rainy and dry seasons, respectively) (Figure 6).

**Figure 7:**
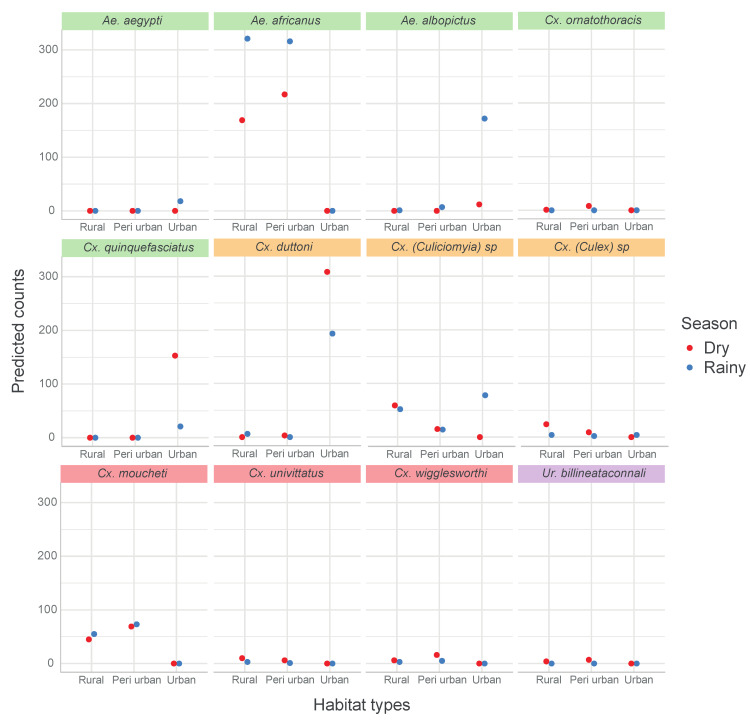
Interaction plot of predicted mosquito species abundance across habitat types and season. Green panel header: mosquito species with significant habitat types and season effects. Orange panel header: mosquito species with significant habitat types:season interaction. Pink panel header: mosquito species with significant habitat types effect only. Purple panel header: mosquito species with a significant season effect only.

The predicted number of mosquitoes (based on negative binomial regression model) for each species per habitat and per season is shown in Figure 7. Only species with a significant effect of either the habitat types, season or their interaction are presented.

*Ae. africanus* were only encountered in peri-urban and rural habitats, and mostly during the rainy season, while *Cx. pipiens* s.l. was only found in urban areas, and in higher quantity in the dry season. On the other hand, *Cx. (Culiciomyia)* spp., *Cx. duttoni* and *Ae. albopictus* were caught in the three habitat types, with significant differences in their abundances in both seasons.

### 3.3. Potential Medical Importance

About 52% of the total mosquito species, subspecies, and species groups collected in this study have been implicated in the transmission of arboviruses (Table 4). These species may be involved in natural arbovirus cycles as principal or secondary epidemic vectors, primary or secondary enzootic vectors, or as incidental vectors with unknown epidemiological importance.

## 4. Discussion

Overall, the number of individual mosquitoes did not vary across habitats and seasons. This suggests little variation in environmental conditions and habitat conditions between seasons in Dschang. The presence of raffia palm bushes in both rural and peri-urban areas may help to provide a year-round humid environment. Such areas with adequate microclimatic conditions for oviposition and reproduction may help a mosquito population persist year-round. Furthermore, the persistence of breeding sites, such as gutters and stagnant water pools, during the dry season in the urban habitat may account for the lack of variation across seasons. On the other hand, the inconsistency of collection methods in the different habitats might have affected the number of collected mosquitoes, while breeding sites investigations were not successful in peri-urban and rural areas (in contrast to the urban area), sweep nets collections of adults were not effective in the urban area (in contrast to peri-urban and rural areas where they were very efficient). These results are different from a study carried out in Andalusia, where a greater mosquito abundance was found in rural areas than in urban areas [72].

Mosquito species richness varied across habitats and seasons. The type of vegetation in the rural and peri-urban areas may account for the observed significant difference in mosquito species richness across habitats. In contra to the urban area, the rural and peri-urban areas are characterised by old forest patches with raffia palms, surrounded by polycultures of vegetables, bananas and other types of fruits. These environmental conditions provide breeding and resting sites and diverse host species for blood meals for the development and survival of many mosquito species [32]. The highest species richness was observed in the peri-urban area and the lowest richness in the urban area. More open areas, such as urban areas (compared to the bushy and forested rural and peri-urban areas), are usually less favourable for the survival of most mosquito species and only a few (e.g., *Ae. aegypti*) have successfully adapted to urban environments. Likewise, in southwestern Cameroon, high mosquito richness was observed in the selectively logged forest, and low richness in the young palm plantation [31]. In other areas, such as central Thailand, urban/suburban habitats were less diverse in terms of mosquito species than forest/fragmented forest habitats [13]. In southeastern Cote d’Ivoire, the greatest species richness was found in the rainforest and the lowest richness in the oil palm monoculture [73]. On the other hand, a higher richness was recorded during the dry season and the lowest richness during the rainy season with more *Culex* species found in the dry seasons. The persistence of mosquito species, as well as their breeding sites, during the dry season indicates that disease transmission could potentially occur at any time of the year in these areas. However, these results are opposed to studies by Steiger et al. [74], Rezaul et al. [75] and Santos et al. [76], who captured more mosquitoes during the wet season than during the dry season.

Importantly, variable mosquito species composition was found among the different habitats and seasons. *Ae. africanus* and *Cx. moucheti* were only found in rural and peri-urban areas, whereas species, such as *Cx. pipiens* s.l. and *Ae. Aegypti,* were only found in the urban area. On the other hand, species from the *Er.* “chrysogaster” group, *Cx. (Culiciomyia)* spp., *Lt. tigripes, Cx. duttoni* and *Ae. albopictus* were caught in all three habitat types. Other species, such as *Ae. aegypti*, *Ae. metallicus, Ae. wellmani* and *Ae. Soleatus,* were only captured during the rainy season and species, such as *Ae. gibbinsi*, *Cx. ornatothoracis*, *Uranotaenia* spp. and *Mimmomyia* spp., were solely trapped during the dry season. To our knowledge, this is the first documented account of the mosquito fauna of Dschang. Located in at least two different habitats, *Ae. africanus Cx. moucheti*, *Cx. (Culiciomyia)* spp., *Er*. “chrysogaster” group, *Lt. tigripes, Cx. duttoni* and *Ae. albopictus* seem to easily adapt to human urban areas. These species can, therefore, be considered as potential ecological bridge vectors that may have the potential to contribute to the transmission of pathogens from rural to peri-urban areas and vice versa. In contrast, *Cx. pipiens* s.l. and *Ae. aegypti* are considered solely urban species in Dschang. *Ae. africanus*, the most abundant species in the study area, predominated in both rainy and dry seasons and in rural and peri-urban areas. The high abundance of *Ae. africanus* is probably related to the vegetational structure of the study area and can be attributed to the presence of raffia palms in rural and peri-urban areas. *Ae. africanus* was also one of the most abundant mosquitoes collected in the raffia palms in Kumbo, Cameroon [77]. These results highlight how raffia palms can provide optimal immature development sites for species that can transmit arboviruses.

At least 19 species collected in this study have previously been implicated in the transmission of a wide variety of arboviruses suggesting a high potential for maintenance and transmission of many arboviruses in the study area, as well as the potential for outbreaks of arboviral diseases in this region. *Ae. africanus*, the most abundant species, can transmit several arboviruses and has been incriminated as a vector of YFV and CHIKV [63]. The broad distribution of this species across rural and peri-urban areas in Dschang suggests the possibility of this species contributing to potential arbovirus outbreaks. *Cx. duttoni*, the second most abundant mosquito species in the study area, has been found to carry arboviruses, such as *Arb11266* [63]. Other mosquito species, although collected in small numbers in our study area, have also been shown to or have been implicated as vectors of many arboviruses. These are *Ae. aegypti* [35], *Ae. circumluteolus* [35,61], *Ae. metallicus* [35], *Ae. simpsoni* [35], *Cx. univittatus* [35], *Ae. argenteopunctatus* [35,56]. Arboviruses that have been found circulating in Cameroon include DENV, YFV, CHIKV, ZIKV, ONNV, NTAV, SPOV, WESV, SFV, SINV, MIDV, WNV, TAHV (Tahyna virus), BUNV, UGSV (Uganda S virus), YAOV, RVFV, BWAV and ILEV [35,40]. More specifically, ZIKV has recently been detected in Cameroon in Yaoundé and in the Fako Division [40]. Furthermore, a recent study found acute dengue in children living in urban and semi-urban areas of Dschang [46]. The potential vectors of these arboviruses in Cameroon include *Ae.* “tarsalis” group (viruses isolated: MIDV, WESV), *Ae. argenteopunctatus* (virus isolated: NKOV), *Ae. africanus* (virus isolated: WESV), *Ae. simpsoni* (virus isolated: WESV), *Cx. (Culiciomyia)* spp. (virus isolated: NTAV), *Cx. moucheti* (virus isolated: NTAV), *Lt. tigripes* (virus isolated: NTAV) and *Er.* “chrysogaster” group (viruses isolated: MIDV, SIMV) [56,59]. The results of these studies and our entomological survey would indicate that an entomological-based arbovirus surveillance system would provide valuable early warning systems to determine the risk of arboviral outbreaks in localities throughout Cameroon. Furthermore, in rural and peri-urban areas, people practice intensive agriculture (as their main source of livelihood) and move from rural and peri-urban areas to urban areas to sell products and for other commercial purposes. This increased flow of people might substantially increase the risk of arbovirus spread and introduction into urban areas.

## 5. Conclusions

This study provides an up-to-date catalogue of the *Culicidae* fauna along a transect of levels of urbanisation and identifies the potential mosquito vectors that may be involved in arbovirus transmission in the West region of Cameroon. Given their presence in at least two habitats, *Ae. africanus, Cx moucheti, Culex (Culiciomyia*), *Cx. duttoni* and *Ae. albopictus* have the potential to act as bridge vectors that can transmit arboviruses from rural to urban areas. The persistence of mosquito species during the dry season suggests the possibility of year-round transmission of arboviruses in the area. Further research is required to assess the prevalence of arboviruses in these areas and to study the arboviral vectorial capacity of these mosquito species as well as to assess their anthropophilic and zoophilic feeding behaviours through blood-meal source identification.

## Figures and Tables

**Table 1:** Total number of mosquito species collected from each habitat type (rural, peri-urban and urban) and seasons (rainy and dry) combination in Dschang, West-Cameroon.

Mosquito Species	Rural			Peri-Urban			Urban			
	Rainy	Dry	Total	Rainy	Dry	Total	Rainy	Dry	Total	Grand-Total
*Ae. argenteopunctatus*	1	0	1	1	0	1	0	0	0	2
*Ae. circumluteolus*	1	0	1	1	0	1	0	0	0	2
*Ae. aegypti*	0	0	0	0	0	0	18	0	18	18
*Ae. africanus*	316	217	533	321	169	490	0	0	0	1023
*Ae. albopictus*	7	0	7	1	0	1	172	12	184	192
*Ae. simpsoni*	0	0	0	1	0	1	1	0	1	2
*Ae. domesticus*	0	0	0	1	0	1	0	0	0	1
*Ae. gibbinsi*	0	0	0	0	5	5	0	0	0	5
*Ae. (Stegomyia)* spp.	1	0	1	1	0	1	0	0	0	2
*Ae. tarsalis*	0	3	3	4	5	9	0	0	0	0
*Ae. wellmani*	2	0	2	1	0	1	0	0	0	3
*Ae. metallicus*	2	0	2	1	0	1	0	0	0	3
*Ae. soleatus*	0	0	0	1	0	1	0	0	0	1
*Ae. fraseri*	0	0	0	1	0	1	0	0	0	1
*An. gambiae* s.l.	0	0	0	0	0	0	0	4	4	4
*Cx. (Culex)* spp.	2	9	11	4	24	28	4	0	4	43
*Cx. (Culiciomyia)* spp.	14	15	29	52	59	111	78	0	78	218
*Cx. invidiosus*	0	1	1	0	0	0	0	0	0	0
*Cx. ornatothoracis*	0	8	8	0	1	1	0	0	0	9
*Cx. duttoni*	0	3	3	6	0	6	193	308	501	510
*Cx. perfidiosus*	0	1	1	0	0	0	0	0	0	1
*Cx. moucheti*	73	69	142	55	45	100	0	0	0	242
*Cx. trifoliatus*	0	0	0	0	1	1	0	0	0	1
*Cx.* spp.	0	1	1	8	2	10	0	3	3	14
*Cx. univittatus*	1	6	7	3	10	13	0	0	0	20
*Cx. wigglesworthi*	5	16	21	3	6	9	0	0	0	30
*Cx. pipiens* s.l.	0	0	0	0	0	0	21	153	174	174
*Cq. annettii*	0	2	2	0	3	3	0	0	0	5
*Cq. maculipennis*	1	4	5	1	5	6	0	0	0	11
*Cq.* spp.	0	0	0	0	3	3	0	0	0	3
*Er.* “plioleucus” gp.	0	0	0	0	1	1	0	0	0	1
*Er.* “chrysogaster” *gp.*	1	2	3	2	4	6	6	0	6	15
*Er.* spp.	0	0	0	0	1	1	0	0	0	1
*Lt. tigripes*	1	9	10	5	21	26	11	20	31	67
*Mi. flavopicta*	0	1	1	0	0	0	0	0	0	1
*Ur. billineata connali*	0	7	7	0	4	4	0	0	0	11
*Ur. mashonaensis*	0	1	1	0	0	0	0	0	0	1
Total	427	376	803	470	373	843	504	500	1004	2650

**Table 2:** Mosquito richness across habitats in Dschang, West-Cameroon.

	Rural Area	Peri-urban Area	Urban Area	*p*
Observed species richness (S)	25	30	11	<0.05
Chao 1	32	121	11	<0.05
ACE	33.37	51.97	11.33	<0.05

**Table 3:** Mosquito richness across seasons in Dschang, West-Cameroon.

	Rainy Season	Dry Season	*p*
Observed species richness (S)	24	28	<0.05
Chao 1	24.6	64	<0.05
ACE	26.04	38.33	<0.05

**Table 4:** Relationship between mosquito species identified in Dschang, West-Cameroon, and some arboviruses.

Mosquito Species	Arboviruses	References
*Ae. argenteopunctatus*	NKOV, SFV, BUNV, MIDV, PGAV, SHOV, WESV, CHIKV, NRIV	[35,56,57,58]
*Ae. circumluteolus*	SPOV, WESV, NDUV, RVFV, BUNV, SIMV, MIDV, GERV, LEBV, PGAV, SHOV, SPOV, WNV	[35,56,57,59,60,61,62]
*Ae. aegypti*	YFV, DENV, ZIKV, CHIKV, RVFV, BUNV, DUGV, ORUV, USUV, VEEV, WNV, SFV, WESV, BBKV	[35,57,60,62]
*Ae. africanus*	YFV, ZIKV, BOUV, BBKV, CHIKV, WESV, WNV, ORUV, BOZV	[35,56,63]
*Ae. albopictus*	DENV, CHIKV, ZIKV, JEV, USUV, YFV	[35,61,62,63,64,65]
*Ae. simpsoni*	YFV, BBKV, NRIV, WESV	[35,56,61,62]
*Ae.* “domesticus” group	WESV, BUNV	[57,62]
*Ae.* “tarsalis” group	MIDV, WESV, KEDV, PGAV, PATV, ZIKV, SHOV, RVFV	[35,56,57,59,62,63,66,67]
*Ae. metallicus*	YFV, ZIKV, WESV	[35,62]
*An. gambiae* s.l.	BUNV, BWAV, CHIKV, ILEV, MIDV, ONNV, ORUV, ZIKV, TATV, NRIV, NDOV, BGIV	[57,68,69]
*Cx. (Culiciomyia*) spp.	NTAV, YAOV, CHIKV, MIDV, BBKV, BGIV	[35,59,62,63]
*Cx. duttoni*	*Arb11266*	[63]
*Cx. moucheti*	NTAV	[59]
*Cx. univittatus*	WNV, USUV, WESV, SINV, RVFV, SPOV	[35].
*Cx. pipiens* s.l.	JEV, WNV, RVFV, USUV, CHIKV, EEEV, KUNV, MTBV, MURV, OROV, RRV, SLEV, SINV, VEEV, WANV, WEEV, WNV, BBKV	[35,57,60,61,62,70,71]
*Cq. maculipennis*	CHIKV	[62]
*Er.* “chrysogaster” group	SFV, MIDV, NTAV, SIMV, NKOV, RVFV	[56,57,59,66]
*Lt. tigripes*	NTAV, KAMV, MOSV, SINV, BBKV	[35,57,59,62,63]
*Ur. mashonaensis*	WESV	[62]

*Abbreviations: Ae. Aedes*; AMTV: Arumowot virus; *An. Anopheles*; BAGV: Bagaza virus; BBKV: Babanki virus; BGIV: Bangui virus; BOUV: Bouboui virus; BOZV: Bozo; BUNV: Bunyamwera virus; BWAV: Bwamba virus; CHIKV: chikungunya virus; *Cx. Culex.*; *Cq. Coquillettidia*; DENV: dengue virus; DUGV: Dugbe virus; EEEV: Eastern equine encephalitis virus; *Er. Eretmapodites*; ILEV: Ilesha virus; JEV: Japanese encephalitis virus; KAMV: Kamese virus; KEDV: Kedougou virus; KUNV: Kunjin virus; *Lt. Lutzia*; MIDV: Middelburg virus; MOSV: Mossuril virus; MTBV: Marituba virus; MURV: Murray Valley virus; NDOV: Nyando virus; NDUV: Ndumu virus; NKOV: Nkolbisson virus; NRIV: Ngari virus; NTAV: Ntaya virus; ONNV: o’nyong-nyong virus; ORUV: Orungo virus; OROV: Oropouche virus; PATV: Pata virus; PGAV: Pongola virus; RRV: Ross River virus; RVFV: Rift Valley fever virus; SFV: Semliki Forest virus; SIMV: Simbu virus; SINV: Sindbis virus; SLEV: St. Louis Encephalitis virus; SPOV: Spondweni virus; TATV: Tataguine virus; USUV: Usutu virus; *Ur. Uranotaenia*; VEEV: Venezuelan equine encephalitis virus; WANV: Wanowrie virus; WEEV: Western equine encephalitis virus; WESV: Wesselsbron virus; WNV: West Nile virus; YAOV: Yaounde virus; YFV: yellow fever virus; ZIKV: Zika virus; *Arb11266 unidentified Flavivirus* related to WNV and Usutu virus, SHOV; Shokwe virus.
